# Increased Frequency of CD4+ CD25+ FoxP3+ T Regulatory Cells in Pulmonary Tuberculosis Patients Undergoing Specific Treatment and Its Relationship with Their Immune-Endocrine Profile

**DOI:** 10.1155/2015/985302

**Published:** 2015-04-19

**Authors:** Ariana Díaz, Natalia Santucci, Bettina Bongiovanni, Luciano D'Attilio, Claudia Massoni, Susana Lioi, Stella Radcliffe, Griselda Dídoli, Oscar Bottasso, María Luisa Bay

**Affiliations:** ^1^Institute of Immunology, School of Medical Sciences, National University of Rosario, Rosario, Santa Fe, Argentina; ^2^Central Laboratory, Centenary Provincial Hospital, Rosario, Santa Fe, Argentina

## Abstract

Tuberculosis (TB) is a major health problem requiring an appropriate cell immune response (IR) to be controlled. Since regulatory T cells (Tregs) are relevant in IR regulation, we analyzed Tregs variations throughout the course of TB treatment and its relationship with changes in immune-endocrine mediators dealing with disease immunopathology. The cohort was composed of 41 adult patients, 20 of them completing treatment and follow-up. Patients were bled at diagnosis (T0) and at 2 (T2), 4 (T4), 6 (T6), and 9 months following treatment initiation. Twenty-four age- and sex-matched healthy controls (HCo) were also included. Tregs (flow cytometry) from TB patients were increased at T0 (versus HCo *P* < 0.05), showing even higher values at T2 (versus T0 *P* < 0.01) and T4 (versus T0 *P* < 0.001). While IL-6, IFN-*γ*, TGF-*β* (ELISA), and Cortisol (electrochemiluminescence, EQ) were augmented, DHEA-S (EQ) levels were diminished at T0 with respect to HCo, with cytokines and Cortisol returning to normal values at T9. Tregs correlated positively with IFN-*γ* (*R* = 0.868, *P* < 0.05) at T2 and negatively at T4 (*R* = −0.795, *P* < 0.05). Lowered levels of proinflammatory cytokines together with an increased frequency of Tregs of patients undergoing specific treatment might reflect a downmodulatory effect of these cells on the accompanying inflammation.

## 1. Introduction

According the World Health Organization one third of the world population is infected with* Mycobacterium tuberculosis* (Mtb), and the majority of affected people are found in developing countries. The situation becomes even more problematical because of the increased susceptibility of HIV infected persons to develop the disease and the emergence of strains resistant to the antimicrobial therapy [1].

Most people infected with Mtb have a clinically latent infection and 10% of them further progress to active TB during their lifetime. Bacterial, host, and environmental factors influence the development of active TB [1, 2]. Commonly, the host immune response (IR) controls Mtb replication, collaborating with the establishment of latent infection, which ultimately depends on a fine balance between the pathogen persistence and the specific IR [3]. T cell-mediated immunity is the main response against TB, mainly through interferon-gamma (IFN-*γ*) production by antigen specific T cells. However an exacerbated effector IR and the ensuing excessive secretion of inflammatory mediators turn out to be detrimental damaging host tissues through immunopathological processes. It follows that a properly balanced IR is essential to successfully cope with this pathogen [4].

Regulatory T cells which tend to modulate the anti-infectious host immunity are currently viewed as one of the suitable mechanisms for Th-1-dependent immune response suppression. Among these T cell subpopulations, those expressing intracellular transcription factor* FoxP3* seem to display the most functional activity in terms of immunosuppression.* FoxP3*-positive regulatory T cells (Tregs) can be either natural, in the course of antigen-dependent thymic differentiation, or induced in the periphery during adaptive immune responses [5]. Tregs represent 5–10% of circulating CD4+ cells but in humans only the subset expressing higher levels of CD25 (*α* chain of IL-2R) exhibits a strong suppressive capacity [6]. The main mechanisms of suppression by Treg cells include inhibition of IFN-*γ* production by T cells through the production of IL-10 and transforming growth factor beta (TGF-*β*), as well as by cell to cell contact [7]. While evidence indicates that Tregs are involved in the immune response against Mtb, there is no consensus regarding the precise role of Treg in TB.

*Some* studies reported Treg cell expansion in the blood, lung, or other tissues of patients with active TB [8, 9], suggesting that these cells may suppress T cell immunity and hence contribute to disease development [6, 10]. In contrast, other studies reported no increase of Treg in TB patients [11, 12]. A recent work carried out in nonhuman primates raised the view of increased Treg as representing a counterbalancing anti-inflammatory response instead of an immunosuppressor effect [7]. Collectively, it may be assumed that Tregs are likely to have a differential role on infection outcomes depending on the proper balance between protective immune mechanisms and those involved with tissue damage.

In parallel, other extrinsic mechanisms may also contribute to reduce inflammation during immunologically based diseases, partly because of the neuroimmunoendocrine communication, in particular the hypothalamus-pituitary-adrenal (HPA) axis, that attempts to maintain immune homeostasis. As key molecules of the HPA axis, endogenous adrenal steroids (Cortisol, Dehydroepiandrosterone (DHEA)) contribute to modulate the immune response to infectious agents and other insults. In turn, the HPA axis is modulated by cytokines like IFN-*γ* and IL-6, which are produced during the immune response. Within this setting, we have observed that patients with pulmonary TB have imbalanced immune-endocrine responses with increased plasma concentrations of proinflammatory cytokines and Cortisol, in presence of decreased DHEA levels associated with disease severity [13, 14].

In expanding the knowledge about the participation of Treg in TB, the analysis of this T cell population throughout the course of TB treatment and its eventual relationship with changes in immune-endocrine mediators involved in disease immunopathology is informative. According to this we have proceeded to characterize this T cell population at different time points following treatment initiation along with the assessment of cytokines and hormones critically to contain an infectious threat.

## 2. Materials and Methods

### 2.1. Subjects

Between January 2010 and January 2014, adults who were diagnosed with lung TB based on clinical and radiological findings and identification of TB bacilli in sputum were enrolled and followed for up to nine months in a prospective cohort study of Mtb infection and treatment. This observational cohort included forty-one patients, with neither HIV coinfection nor multidrug resistant TB. Patients had mild (*n* = 7), moderate (*n* = 16), or advanced (*n* = 18) disease according to the radiological findings, as previously described [13]. Antituberculosis therapy consisted of six months of rifampicin and isoniazid, initially supplemented by two months of pyrazinamide and ethambutol. Of the 41 recruited patients, blood samples at all time points were available in 20 of them (mild = 1, moderate = 12, and severe = 7). Twenty-four matched healthy subjects (HCo), living in the same area and without antecedents of contact with TB patients, were included as controls. Exclusion criteria for all participants included pathologies affecting the hypothalamus-pituitary-thyroid- or gonadal-axis, or direct compromise of the adrenal gland, pregnancy, contraceptive drugs, age under 18, or systemic or localized pathologies requiring treatment with corticosteroids or immunosuppressants. All subjects were BCG vaccinated and provided written consent to participate in the study. The study was approved by the Ethical Committee of the Facultad de Ciencias Médicas, Universidad Nacional de Rosario and Centenario Hospital of Rosario.

### 2.2. Sample Collection

Blood samples were obtained from TB patients at the time of diagnosis (before initiation of the treatment, T0) and 2, 4, and 6 months (T2, T4, and T6) after starting the specific antituberculosis treatment. An additional sample was obtained three months after the end of treatment completion (T9). All samples were obtained between 8:00 and 9:00 a.m. with and without EDTA and then centrifuged. Aprotinin (100 U/mL; Aprotinin from bovine lung, Sigma-Aldrich, St. Louis, MO, USA) was added to the plasma shortly after collection and the samples were preserved at −20°C. Serum samples were immediately processed for hormone quantification. One blood sample was obtained from age- and sex-matched HCo and processed in the same way.

### 2.3. Sample Preparation

Twenty milliliters of blood was obtained using EDTA as anticoagulant, and the PBMC were obtained by Ficoll-Hypaque density gradient centrifugation (Biowittaker, Walkersville, MD). PBMC were washed twice in PBS and counted by trypan blue staining in a Neubauer chamber. Viability was always of 95%.

### 2.4. Flow Cytometry

For surface marker analyses, a sample of 50 *μ*L of well-mixed EDTA anticoagulated whole blood was incubated for 30 min at room temperature with 20 *μ*L of BD Tritest CD4 FITC/CD8 PE/CD3 PerCP Reagent (BD Biosciences, San Jose, CA, USA) according to manufacturer instructions. Following incubation, red blood cells were lysed with NaHCO_3_/ClNH_4_ solution. Cells were washed twice with PBS and preserved for cytometric analysis.

Another sample of 1 × 10^6^ PBMC was incubated for 30 min with anti-CD4-FITC and anti-CD25-PEcy5.5 (BD Biosciences, San Jose, CA, USA) and for intracellular staining of FoxP3, the cells in the tubes were resuspended in Fix/Perm buffer (eBioscience, San Diego, CA, USA) and left at 4°C for 30 min. Subsequently, these cells were washed with cold PBS and with permeabilization buffer (eBioscience, San Diego, CA, USA) and then 20 *μ*L anti-human FoxP3-PE (BD Biosciences, San Jose, CA,USA) was added and the cells were incubated at 4°C for 30 min. Stained cells were analyzed with a FACSAria II flow cytometer (BD Biosciences, San Jose, CA, USA). The percentage of positive cells and the mean fluorescence intensity (arbitrary units) for a specific marker were calculated using FACSDiva software (BD Biosciences, San Jose, CA, USA). For purpose analysis 30000 events were recorded.

### 2.5. Evaluation of Immunological Mediators and Hormones

Levels of TGF-*β*, IFN-*γ*, and IL-6 were measured in plasma using commercially available high-sensitivity ELISA kits according to the instructions of the manufacturer. Detection limits were 0.07 pg/mL for IL-6 (R&D Systems, Inc. Minneapolis, USA), 125 pg/mL for TGF-*β*, and 4.7 pg/mL for IFN-*γ* (both from BD Bioscience, San Jose, CA, USA). Cortisol and DHEA-sulphated (DHEA-S) serum concentrations were assessed by electrochemiluminescence (Cobas e411, Roche, Germany).

### 2.6. Statistical Analysis

Comparisons between groups (TB versus HCo) were performed by nonparametric methods, (Kruskall-Wallis analysis of variance and Mann-Whitney *U* test). Correlations between variables were assessed by Spearman's rank test. Data were considered statistically significant when *P* < 0.05. The analyses were performed using GraphPad Prism 4 Software.

## 3. Results

### 3.1. CD4+CD25+FoxP3+ T Cells Are Increased in the Blood of Patients with TB

**Figure 1:**
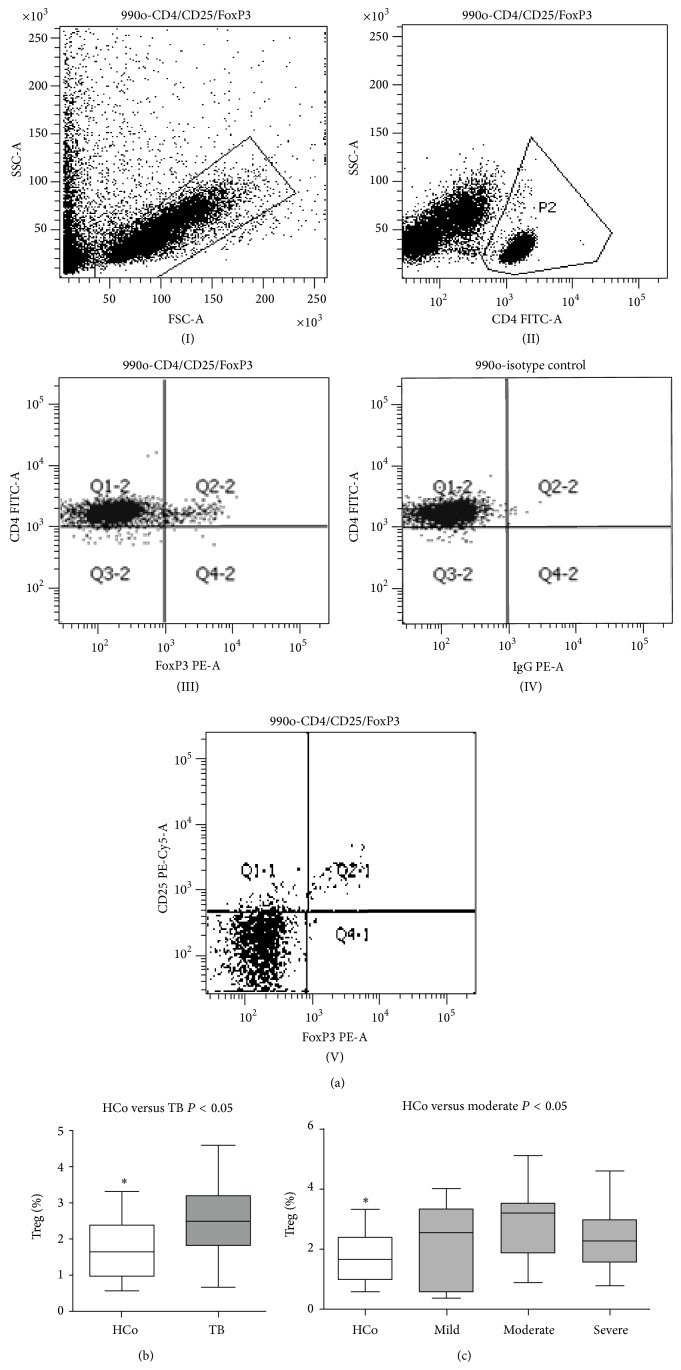
CD4+CD25+FoxP3+ T cells (Tregs) frequency in TB patients. (a) Plot of cytometric analysis from a representative sample: (I) FSC and SSC identifying mononuclear cells, (II) CD4+ cells within the lymphocyte gate, (III) assessment of FoxP3+ cells gated on the CD4+ cells as shown in panel II, (IV) isotype control from the same sample, and (V) an additional control showing that the CD4+FoxP3+ population is also CD25 high. (b) Treg % comparisons between HCo and TB. (c) Comparisons of disease severity of TB patients. Box plots show median values, 25–75 percentiles of data in each group with maximum and minimum values. HCo: healthy controls.

Table 1 shows some general characteristics of study groups. All patients showed a decreased Body Mass Index (BMI) (*P* < 0.001). There were no differences with respect to age, sex distribution, BCG-vaccination, and peripheral CD8+ T cells frequency. However, we found that TB patients presented a significant diminution in CD4+ T cell levels (*P* < 0.05). Then we investigated whether active TB was associated with a CD4+CD25+FoxP3+ Treg expansion by assessing their frequency in PBMC of TB patients at the time of diagnosis by flow cytometry (Figure 1(a)). Results depicted in Figure 1(b) indicate that the frequency of Treg cells within the total CD4+ population was significantly increased in TB patients compared with HCo (*P* < 0.05). We next analyzed the percentage of Treg cells in TB patients according to their severity. As seen in Figure 1(c), moderate TB patients differed from HCo.

### 3.2. Treg Frequency during Antituberculosis Therapy

**Figure 2:**
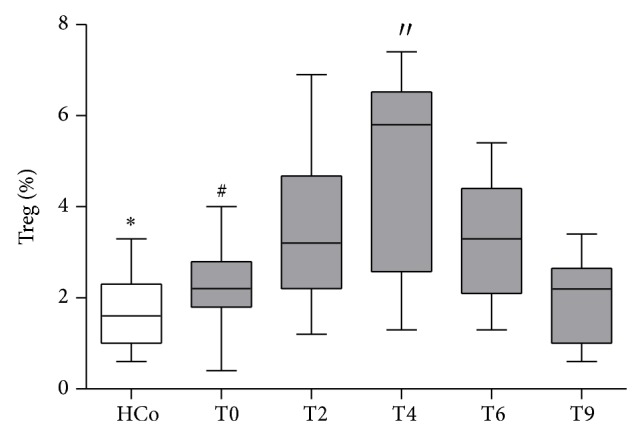
Frequency of CD4+CD25+FoxP3+ T cells (Tregs) within the total CD4+ population of TB patients throughout treatment. Box plots show median values, 25–75 percentiles of data in each group with maximum and minimum values. HCo: healthy controls. T0: time of diagnosis; T2, T4, T6: 2, 4, and 6 months after starting the specific anti-TB treatment; T9: 3 months after the end treatment completion. ^*^HCo versus T0 and T6 *P* < 0.05; HCo versus T2 and T4 *P* < 0.01. ^#^T0 versus T2 *P* < 0.05; T0 versus T4 *P* < 0.01; ′′T4 versus T9 *P* < 0.05.

In earlier studies, we have evaluated immune and endocrine parameters during TB treatment, showing that certain cytokines were augmented at the time of diagnosis (IL-1*β*, IL-6, and IFN-*γ*) and even 2 months after treatment initiation (IFN-*γ*) [15]. In relation to peripheral T cells, we found a decrease in the percentages of CD3+ CD4+ cells at the time of diagnosis that attained normal values upon starting specific treatment (data not shown). When assessing the frequency of Tregs throughout specific treatment, these cell populations remained significantly increased during that period compared to values in HCo, reaching their highest relative levels at T4 (HCo versus T4 *P* < 0.01, Figure 2).

### 3.3. Correlations between Treg Frequency and Cytokine and Hormone Levels

TGF-*β*, IFN-*γ*, and IL-6 plasma levels were assessed at the beginning and at different time points during anti-TB treatment.  Table 2 shows median values and interquartile range for each cytokine at the study time points. The three cytokines were increased at the time of diagnosis with respect HCo, returning to normal values once patients initiated specific treatment. Pairwise correlations between cytokines and Treg frequency revealed that IFN-*γ* levels were positively associated with % Treg at month two under treatment (*R* = 0.868, *P* < 0.05, *n* = 8). Notably, this correlation became negative when patients underwent four months of specific treatment (*R* = −0.798, *P* < 0.05, *n* = 9; see Supplementary Material available online at http://dx.doi.org/10.1155/2015/985302). In a further step TB patients were categorized according to their IFN-*γ* levels at T4. In this way patients with values below the median value had significantly higher levels of Treg cells when compared with cases whose plasma IFN-*γ* is situated above the median concentration (*P* = 0.027).

With regard to adrenal hormones, serum Cortisol was increased at the time of diagnosis (*P* < 0.01) and at T2 (*P* < 0.01), reaching values comparable to HCo at T4. DHEA-S levels which were found decreased at T0 (versus HCo *P* < 0.05) remained so during the whole treatment period but were augmented at T9 reaching values similar to those seen in HCo. Unlike this DHEA-S levels which were found strongly decreased at diagnosis started to increase earlier following treatment initiation (data not shown). Both adrenal steroids showed a negative correlation with the frequency of Treg cells, DHEA-S at the time of diagnosis (*R* = −0.51, *P* < 0.04, *n* = 13), and Cortisol at T4 (*R* = −0.504, *P* < 0.05, *n* = 12; see Supplementary Material).

## 4. Discussion

The involvement of Treg cells in the control of immune responses to self-antigens and in immune homeostasis is well established. Also there is increasing evidence for a role of Treg in the regulation of immunity to infection [16]. It is believed that Treg downmodulate the IR after pathogen eradication to avoid exacerbated pathology. Although this mechanism benefits the host during acute infections, it may be worrying in the chronic setting, notably when pathogen persistence is sustained in spite of an active IR [10]. During chronic infections, a complex interplay is established between the IR to the infectious agent and the ability of the microorganism to evade this protective IR. In TB, a predominant Th1 cellular response is necessary to effectively eradicate the mycobacterium. However if this response exacerbates, certain regulatory mechanisms would be activated to downmodulate it with the aim of safeguarding the affected organ, at the expense of some interference with pathogen clearance. One of these regulatory mechanisms involves Treg [5]. CD4+CD25 high T cells have been reported to be present at elevated levels in TB and to be able to depress T-cell-mediated IFN-*γ* production in these patients [4, 8, 9, 17]. Here, we show that TB patients have an increased frequency of CD4+CD25+FoxP3 Treg in comparison with the group of healthy donors. Furthermore, the frequency remained augmented at two and four months of treatment commencement, reaching values comparable to those seen HCo once the treatment was completed. Tregs are known to display a diverse range of Treg-mediated immunological effects, in terms of antimicrobial and tissue-damage protection. In this case, the increased presence of Treg cells seems to be more compatible with an attempt to downmodulate immunological damage, considering our former [14, 18] and present demonstrations wherein advanced TB coexisted with increased levels of circulating proinflammatory cytokines.

The role of Tregs in TB is not well understood; and it is unclear whether their expansion is a cause or a disease consequence. As stated, they are probably expanded as an adaptive host response to limit the inflammatory reaction and tissue damage induced. Tregs expansion during Mtb infection upon recognition of particular bacterial antigens seems to be achieved by the presence of IL-10 and TGF-*β*, which are known to be highly produced in patients with active TB [14, 17, 19, 20]. Some studies reported a higher suppressive capacity of Tregs on IFN-*γ* producing cells with respect to those producing IL-17, which allowed the persistence of the latter ones in inflamed tissues and thus the perpetuation or recrudescence of inflammation [6, 21, 22]. Our results about a significant positive correlation between Tregs and IFN-*γ* plasma levels at two months of treatment suggest that increased Tregs are not downmodulating inflammation, at this stage. However at T4 when the clinical improvement was quite evident, this correlation turned out to be negative. It follows that month 4 might represent the best time when a proper balance between Treg functioning and clinical response is established.

The negative correlations between adrenal steroids and the frequency of Treg cells are also worth discussing. Glucocorticoids are potent anti-inflammatory and immunosuppressive agents, playing a role in the feedback inhibition of immune/inflammatory responses to maintain homeostasis. Xiang and Marshall Jr. demonstrated that after 24 h of culture of PBMC with dexamethasone (10^−8^ M) FoxP3 mRNA was significantly decreased, suggesting a rapid sensitivity of Treg to corticosteroids [23]. In the same sense, DHEA was also found to inhibit FoxP3 expression in HIV-coinfected TB patients [24]. In line with these* in vitro* findings, in our patients the frequency of Treg cells was inversely correlated with the amount of adrenal steroids implicating an extra loop of influence on this T cell population which is beyond the intrinsic immunological components.

Ribeiro-Rodrigues et al. showed that frequencies of Treg were increased in PBMC of patients with active tuberculosis and did not decline at the end of 6 months of curative antibiotic therapy. At this time point, Treg still preserved their capacity to suppress IFN-*γ* production [25]. Chen et al. evaluated Treg frequencies in pulmonary tuberculosis patients after 2 years of chemotherapy and found a direct correlation between the decreased levels of Treg toward normality in patients successfully recovered, as seen in our case. This group also found that Tregs from tuberculosis patients not only suppress IFN-*γ* production but also inhibit IL-10 production by CD4+ T cells [26].

To conclude, the persistent increase of Tregs until 4 months of specific treatment in presence of diminished amounts of proinflammatory cytokines along with the inverse association between both immune parameters, lend some support to the view of a damage-protective function of Tregs in TB. Further functional studies are now being planned to assess the validity of the assumption of Tregs as counterbalancing the excessive inflammatory response encompassing this disease.

## Supplementary Material

Supplementary material provides data about the correlations between the frequency of Tregs and IFN-γ as well as adrenal steroids at different time points during specific treatment

## Figures and Tables

**Table 1:** Characteristics of study groups.

	HCo (*n* = 24)	TB (*n* = 41)	*P* value
Age (years)^*^	50.5 (25.8–57.3)	45.0 (21.5–54.8)	ns
Sex (F/M)	1/23	4/37	ns
BCG (%)	95%	80%	ns
BMI (Kg/m^2^)^*^	27.4 (25.1–30.8)	19.9 (18.3–23.8)	*P* < 0.001
%CD4+^*^	51.4 (43.0–55.1)	39.4 (32.6–52.6)	*P* < 0.05
%CD8+^*^	33.0 (24.6–35.8)	25.2 (21.8–33.4)	ns

^*^Results are shown as median values (25–75 percentiles). HCo: healthy controls.

**Table 2:** Cytokine and hormone levels in TB patients during specific treatment.

Cytokines and adrenal steroid hormones	HCo group	TB group				
		T0	T2	T4	T6	T9
IL-6 (pg/mL)	1.05^a^ (0.4–2.2)	12.11^a§^ (5.7–23.9)	4.7^a^ (1.8–11.0)	2.9 (1.7–3.6)	3.1 (1.6–3.6)	3.1 (1.0–4.7)
IFN-γ (pg/mL)	4.7^b^ (47–8.2)	13.6^b#^ (10.7–24.3)	6.9^b^ (5.2–12.5)	6.1^b^ (4.7–10.7)	4.7 (4.7–69)	4.7 (4.7–7.2)
TGF-*β*	572.1^c^ (531.3–592.4)	688.5^c*ξ*^ (591.6–804.0)	629.0 (573.3–748.1)	597.5 (519.0–728.3)	597.0 (552.4–692.4)	593.2 (529.6–683.6)
Cortisol (*μ*g/dL)	14.6^d^ (12.3–16.3)	22.3^dΥ^ (15.8–28.1)	19.6^d^ (15.6–21.3)	16.6 (11.4–21.9)	18.1 (12.6–20.6)	16.4 (13.1–20.9)
DHEA-S (*μ*g/dL)	200.0^e^ (133.4–233.9)	129.5^e†^ (60.5–183.5)	112.8^e^ (58.4–180.7)	125^e^ (57.7–165.0)	120.2^e^ (80.15–197.0)	204 (130.3–334.5)

Comparisons between groups.

^a^HCo versus T0, *P* < 0.01, versus T2, *P* < 0.05; ^b^HCo versus T0, *P* < 0.01, versus T2, *P* < 0.05, versus T4, *P* < 0.05; ^c^HCo versus T0, *P* < 0.05; ^d^HCo versus T0, *P* < 0.01, versus T2, *P* < 0.05; ^e^HCo versus T0, *P* < 0.01, versus T2, *P* < 0.05, versus T4, *P* < 0.05, versus T6, *P* < 0.05.

Comparisons within TB patients.

^§^T0 versus T2, T4, T6, T9, *P* < 0.05; ^#^T0 versus T2, T4, T6, T9, *P* < 0.05; ^*ξ*^T0 versus T2, T4, T6, T9, *P* < 0.05; ^Υ^T0 versus T9, *P* < 0.05; ^†^T0 versus T9, *P* < 0.05.

Results are shown as median values (25–75 percentiles). HCo: healthy controls. T0: time of diagnosis; T2, T4, T6: 2, 4, and 6 months after starting the specific anti-TB treatment; T9: 3 months after the end treatment completion. Comparisons for independent groups were made by the Kruskall-Wallis analysis of variance followed by post hoc test when applicable. Comparisons for related samples were performed by the Friedman analysis for variance.
