# Supplementary material for: Increased Frequency of CD4+ CD25+ FoxP3+ T Regulatory Cells in Pulmonary Tuberculosis Patients Undergoing Specific Treatment and Its Relationship with Their Immune-Endocrine Profile
Source: J Immunol Res. 2015 Apr 19;2015:985302. doi: 10.1155/2015/985302 (PMC4417597; doi:10.1155/2015/985302)
Supplement: Supplementary file 1 — Supplementary material provides data about the correlations between the frequency of Tregs and IFN-γ as well as adrenal steroids at different time points during specific treatment [file 985302.f1.pdf]

Correlations between Treg frequency and cytokine and hormone levels

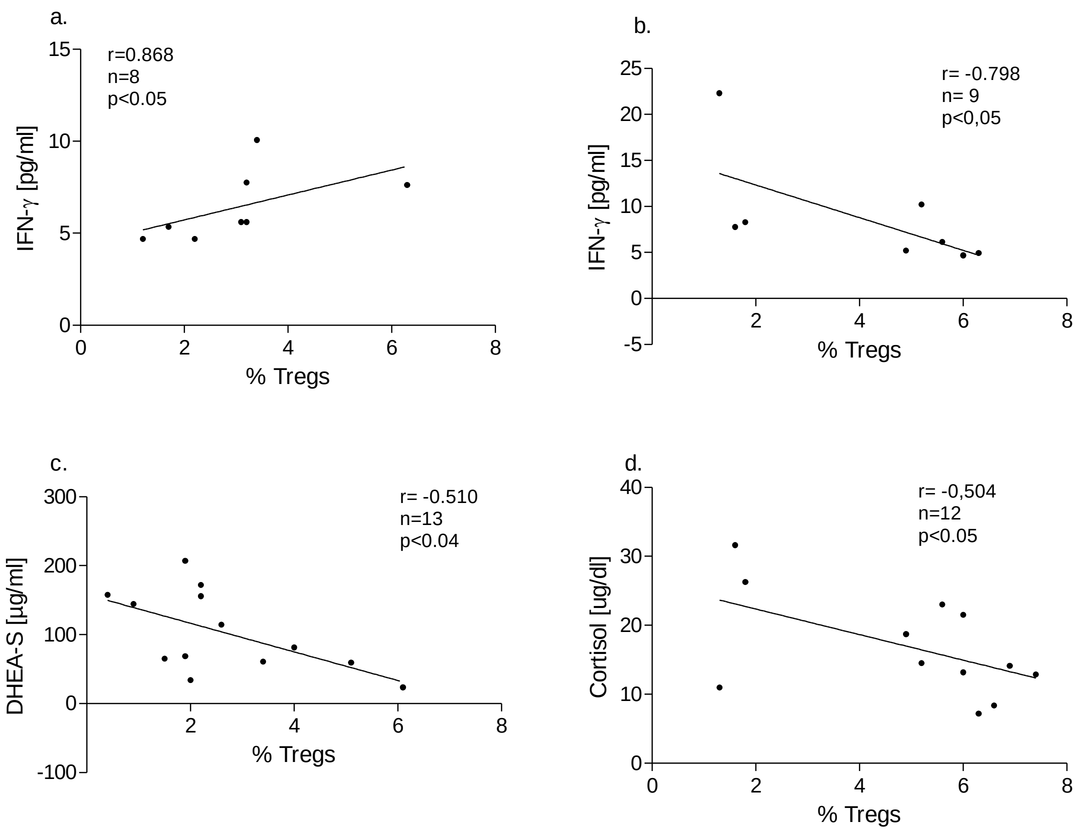

Correlations between Treg frequency and levels of IFN-γ at T2 (a) and T4 (b), DHEA-S at T0 (c) and Cortisol at T4 (d).
